# Supplementary material for: Are carbon emissions trading and green financial instruments synergistic? -Comprehensive quantitative research based on content analysis
Source: PLoS One. 2024 Mar 7;19(3):e0298601. doi: 10.1371/journal.pone.0298601 (PMC10919601; doi:10.1371/journal.pone.0298601)
Supplement: S1 Data — (ZIP) [file pone.0298601.s001.zip › Data/地级市Z_F工作报告.zip!/╣π╬≈/╟╒╓▌╩╨/└┤╫╘╣1⁄2╓┌║┼í╛┬φ┐╦╩2╛▌═°í┐_2018.docx]

各位代表：

　　现在，我代表市人民政府向大会报告工作，请予审议，并请各位政协委员和其他列席会议的同志提出意见。

　　一、2017年工作回顾

　　过去一年，在以习近平同志为核心的党中央英明领导下，我们认真贯彻落实党中央国务院、自治区党委政府和市委各项决策部署，坚持稳中求进的工作总基调，深入实施“创新年”活动，积极稳妥应对经济下行压力和各种困难挑战，稳增长、促改革、调结构、惠民生、防风险等各项工作扎实推进，全市经济社会继续保持平稳健康发展的好势头。

　　（一）紧抓发展第一要务不动摇，推动经济稳步向好取得新成效。预计多项主要经济指标增速居全区前列，地区生产总值增长8.8%；规模以上工业总产值、规模以上工业增加值分别增长24.8%、12.3%；固定资产投资增长14.5%；一般公共预算收入增长7.8%；港口吞吐量增长19.9%，其中集装箱吞吐量增长28.8%；社会消费品零售总额增长10%；外贸进出口总额增长11.3%；城镇、农村居民人均可支配收入分别增长7%、8%。

　　（二）释放港口发展潜力，打造南向主通道初显新效应。中新互联互通南向通道正式开通运营，“渝桂新”海铁联运班列实现常态化运行，“陇桂新”“蓉欧+”东盟国际海铁联运班列贯通，北部湾港至印度/中东远洋航线和至香港、新加坡公共航线天天班开通。大榄坪港口发展区总体规划成功纳入广西北部湾港总体规划和自治区海洋功能区划修编，10万吨级集装箱双向航道一期、金鼓江16－17#泊位开工建设，金鼓江13#泊位等项目加快推进，建成钦州港东站货场扩建一期、30万吨级支航道、鹰岭作业区监管设施等，通关一体化改革全面覆盖进出口领域，新增12个泊位对外开放，柳州汽车“南车北运”海上通道、中泰水果快线启动运营，新开通钦州港至泰国越南印尼等5条集装箱航线，集装箱远洋航线实现零的突破。

　　（三）发力重大项目建设，临港产业链延伸实现新突破。总投资228亿元的华谊化工新材料一体化基地开工，这是继中石油千万吨炼油厂后我市又一个投资超200亿元的重大工业龙头项目，全年引进亿元以上项目150个，新开工500万元以上项目2669个、增长10.8%。百万吨改性沥青一期竣工，百万吨乙烷制乙烯、福佳丙烷综合利用等项目前期工作加快推进，百万吨芳烃项目具备开工条件，钦州石化园区连续5年荣膺全国化工园区20强。中船修船项目竣工，启动了2万吨级造船生产线；卓能新能源锂电池制造一期投产，列入工信部符合《汽车动力蓄电池行业规范条件》企业目录，成为五菱、北汽、东风等新能源汽车企业供应商；力顺5万辆轻型载货汽车项目竣工并获工信部轻型车生产资质。临港产业集群快速发展，中石油、国投电厂等企业生产稳定，新天德能源、天恒石化等企业扩大产能并延伸产业链，一批投资超10亿元项目建设蹄疾步稳，金桂二期180万吨白卡纸等项目前期顺利推进，临港规模以上工业总产值增长36.3%。

　　（四）激发改革动力平台优势，深化开放合作展现新气象。深入推进供给侧结构性改革，出台进一步降成本24条措施，29家企业获准参与电力直接交易，减少用电成本超1亿元；实施购买新建商品房契税补贴等措施，房地产库存比上年末减少28.4%。引进光大银行设立分支机构，新增“新三板”挂牌企业1家，正式开展政策性融资担保业务，金融业进一步脱虚向实。组织实施园区基础设施项目112个，中央预算内投资项目按进度完成。有效实施重点领域改革，取消和调整行政审批事项332项，清理规范和取消行政审批中介服务事项56项，对外公布市直部门行政许可事项373项，正式实施“36证合一”工商登记模式；市行政审批局设立运行，全面实现“一枚公章管审批”。全力推进联合办税，我市获“全国百佳国税地税合作市级示范区”称号。充分发挥国家级平台作用，中马钦州产业园区成为全区首个实行法定治理机构模式园区，鑫德利光电科技一期、凯利数码一期等多个跨国产业链项目建成投产，23个重大产业项目全面建设，马来西亚创新城等项目落户，获批设立海峡两岸产业合作区钦州产业园。钦州保税港区建成全国首个进口酒类综合服务产业知名品牌示范区，获批运行进境水果指定口岸，成为全区首批CEPA先行先试示范基地。中国－东盟港口城市合作网络被确定为中国－东盟交通合作重点项目，组建了中方秘书处，合作网络成员增加到24个，海上搜救分中心等4个航运配套服务项目基本建成。

　　（五）推动区域协调发展，城乡统筹迈出新步伐。围绕壮大县域经济，出台县域经济发展“1+4”系列政策文件，钦南区荣获“广西科学发展十佳县”，陆屋机电产业园等项目投产运营，县域规模以上工业总产值增长19.5%；完成年度粮食生产任务，“双高”糖料蔗基地加快建设，农村土地承包经营权确权登记可颁证率97.2%，钦州港大蚝养殖等7个现代特色农业示范区获认定为自治区级核心示范区，钦州大蚝上榜“2017中国百强农产品区域公用品牌”，钦州蚝情节获评为国家级示范性渔业文化节庆，“三品一标”认证产品达177个，总数全区第一；创新发展农村电商，浦北县获评为“广西农村淘宝优秀县域”；积极推进全域旅游，三娘湾渔村获评为国家级“最美渔村”，灵山县苏村获评为“中国美丽休闲乡村”，浦北县获认定为全国第7个“世界长寿之乡”，钦北区那桑村获评为“中国少数民族特色村寨”，园博园、林湖公园、六峰山、大芦村、越州天湖成功创建4A级景区。全面建设滨海新城“三场两街一带一湾”，新城吾悦广场等项目开工建设，疍家风情小镇等项目竣工；城市主干路网加快建设，北部湾大道至中马钦州产业园区城市道路开工建设，金海湾西大街扩建、北部湾大道二期建成通车；加强城市饮用水安全保障，郁江调水工程开工建设；坭兴陶文化创意产业园等3个集聚区列入自治区首批现代服务业集聚区；大力推进全国智慧城市试点，华为云计算及大数据中心过渡机房建成运营。贵港至合浦高速公路、陆屋至灵山一级公路等高等级公路建成通车，实现了县县通高速公路；灵山县、浦北县成功创建“国家园林县城”，陆屋镇列入第二批全国特色小镇，“宜居乡村”四个专项活动深入开展，城乡环境有效改善。

　　（六）推动社会事业改革创新，全力补齐民生短板上有新作为。社会保障体系日趋完善，城镇新增就业、农村劳动力转移就业超额完成任务，在全区率先完成城乡居民医疗保险制度整合，实现异地就医住院费用直接结算，药品零差率销售实现全覆盖。农村危房改造、保障性安居工程建设扎实推进，城乡低保补助标准提高。“互联网+警务”模式获全国推广，特殊监区医院建成使用，社会治安防控体系进一步完善，创新建立信访接访平台，有效预防和化解社会矛盾。安全生产形势总体稳定，食品药品监管科学化水平和突发事件应急联动能力有效提升，创建国家食品安全示范城市有序开展。全面建立“河长制”，加快中央环保督察、国家海洋督察、国家林业督察等反馈意见整改落实，36座镇级污水处理厂建成试水，完成自治区下达的各项环保目标任务。民生支出累计完成170.4亿元，占一般公共预算支出比重83%。脱贫攻坚扎实开展，易地扶贫搬迁项目建成入住，实现57个贫困村摘帽、3.56万贫困人口脱贫。新建、改扩建公办幼儿园44所、公办中小学校9所，有效缓解入园入学难问题，筹建北部湾大学通过自治区检查评估，正式上报教育部。高新区联创科技企业孵化器、浦北裕源药业技术中心通过自治区认定，新增13家高新技术企业，高新技术企业产值增长160%。坭兴陶人才小高地成为第七批自治区级人才小高地。建成使用市图书馆新馆和刘永福纪念馆，组织开展纪念刘永福诞辰180周年系列活动，成功举办市第六届运动会，竞技体育、全民健身蓬勃开展。坚持计划生育基本国策，不断提升计生服务管理水平。

　　民族、宗教、外事、侨务、供销、统计、贸促、人防、海防、科普、气象、水文、档案、决策咨询、地方志、哲学社会科学等工作取得新进展，海关、检验检疫、海事、边检、消防、税务、人民银行、银监、供电、烟草、通信、邮政、盐务等为我市经济社会发展作出了新贡献，妇女儿童、残疾人、老龄和关心下一代等事业取得新进步，国防教育和后备力量建设深入开展，边海防基础设施建设进一步加强，双拥共建工作取得新成绩，民族团结进步创建活动扎实推进。

　　各位代表，过去一年，我市大事喜事接踵而至。中新互联互通南向通道实现开通运营，钦州在构建“一带一路”国际陆海贸易新通道进程中赢得了主动。上海华谊斥资二百亿布局钦州，打造世界化工新材料标杆工程，北部湾现代化大型修造船基地建设取得突破性进展，石化、装备制造两个千亿元产业发展基础进一步夯实。成功承办2017中国－东盟女企业家创业创新论坛、世界沙滩排球巡回赛钦州公开赛、首届环广西公路自行车世界巡回赛等国际性活动，进一步提升了钦州的影响力和美誉度。

　　各位代表，过去一年，我们深入宣传贯彻党的十九大精神，始终以习近平总书记系列重要讲话精神和治国理政新理念新思想新战略为遵循，以习近平总书记赋予广西“三大定位”新使命和“五个扎实”新要求统领工作全局，坚持科学理政、依法行政、从严治政，推动“两学一做”学习教育常态化制度化，持续加强政府自身建设。进一步完善决策程序，认真执行市人大及其常委会的决议、决定，自觉接受人大的法律监督、工作监督和政协的民主监督，认真听取各民主党派、工商联、无党派人士和各人民团体意见建议，积极回应人大代表、政协委员和社会各方面的关切，77件人大代表建议、315件政协提案全部办复，满意率分别为97.4%和100%。全面推行政务公开，强化行政监察和经济责任审计，严控“三公”经费支出。全面落实中央八项规定精神、国务院“约法三章”和党风廉政建设“一岗双责”，加强扶贫领域监督执纪问责，严厉整肃庸政懒政怠政行为，坚决查处“四风”和腐败问题，政府队伍建设取得新成效。

　　各位代表，过去一年，我们紧紧抓住发展第一要务，精准发力，顽强拼搏，取得的成绩来之不易。这是党中央国务院亲切关怀、自治区党委政府和市委坚强领导的结果，是市人大、市政协和社会各界大力支持和有效监督的结果，是广大干部群众凝心聚力、团结奋斗的结果。我代表市人民政府，向广大干部群众，向人大代表、政协委员、各民主党派、工商联、各人民团体和离退休老同志，向驻钦人民解放军、武警官兵和中直、区直单位，向参与和支持钦州发展的各界朋友，表示衷心的感谢和崇高的敬意！

　　肯定成绩的同时，我们也清醒看到发展中存在的困难和问题：产业不强结构不优，产业集聚度不高、产业链不长，新兴产业尚未形成规模；部分重大项目推进困难、落地受限，拉动作用和支撑力不足；发展不平衡不充分的问题突出，发展的质量和效益不高，财政增收乏力，收支矛盾凸显，风险防控压力增大；国家级平台作用发挥不够，开放水平有待提高；县域经济综合实力不强，城乡发展不均衡不协调，民生社会事业存在不少短板；土地、海域等发展要素瓶颈约束进一步趋紧，生态环境保护与节能减排任务艰巨；庸政懒政怠政、不作为、乱作为、消极腐败现象依然存在，营商环境优势相对弱化。对这些问题，我们必须勇于面对，深刻反思，对症施策，下更大的决心、想更多的办法、用更大的力度加以解决。

　　二、2018年工作目标和主要任务

　　今年是贯彻党的十九大精神的开局之年，是改革开放40周年、自治区成立60周年，也是决胜全面建成小康社会、实施“十三五”规划承上启下的关键一年，做好今年政府工作意义重大。党的十九大为中国特色社会主义新时代新征程绘就新蓝图，“一带一路”建设特别是中新互联互通南向通道建设深入推进，为我市经济社会跨越发展增添了新机遇新动力。我们要主动融入新时代，切实担当新使命，抢抓机遇谋发展，聚焦产业、立足生态、着力富民，努力实现更高质量更高效益的发展。

　　今年政府工作的总体要求是：高举习近平新时代中国特色社会主义思想伟大旗帜，全面贯彻落实党的十九大精神，落实“三大定位”新使命和“五个扎实”新要求，坚持稳中求进工作总基调，坚持新发展理念，紧扣社会主要矛盾变化，抢抓多重叠加战略机遇，践行以人民为中心的发展思想，以稳增长为主题、以供给侧结构性改革为主线、以提高发展质量和效益为目标，以实施“服务企业年”为载体，力促经济社会持续健康发展，为建设“一带一路”南向通道陆海枢纽城市，加快构建大开放、大通道、大港口、大产业、大物流新格局打下坚实基础。

　　今年经济社会发展的主要预期目标是：地区生产总值增长8%左右，规模以上工业总产值增长18%左右，规模以上工业增加值增长11%左右，固定资产投资增长12%左右，一般公共预算收入增长8%左右，港口吞吐量突破9000万吨，集装箱吞吐量突破200万标箱，外贸进出口增长10%左右，社会消费品零售总额增长10%左右，城镇居民人均可支配收入增长8%，农村居民人均可支配收入增长9%。

　　（一）充分释放海的潜力发展向海经济，着力建设现代化经济体系。面向海洋发展，向海洋发掘资源和财富，既是我们的独特优势，也是我们实现赶超跨越的必然选择。依托港口资源大力发展石化、能源、海洋工程、装备制造等现代临港产业体系，全力支持实体经济发展，努力提高经济发展质量和效益。

　　集群发展临港产业。围绕延伸石化产业链，建成运营30万吨级油码头，建成金鼓江13#泊位、加快建设16－17#泊位、开工建设12#及14－15#泊位，启动建设三墩循环经济示范岛，加快华谊化工新材料一体化基地等项目建设，开工建设百万吨芳烃一期、新天德乙醇深加工等项目，推进玉柴石化30万吨异辛烷等项目投产、钦州至南宁成品油管道按计划投产运行，开展精细化工产业园、百万吨乙烷制乙烯、福佳丙烷综合利用等项目前期工作，争取引进新浦凝析油综合利用等重大石化项目，加快打造千亿元国家级石化产业基地。推动中船2万吨级造船生产线、集装箱CFS项目开工建设，积极建设北部湾大型修造船基地、新能源轻型载货汽车生产基地。开工建设金桂二期180万吨白卡纸等项目，促进造纸、新材料、新能源等产业集群发展壮大。

　　升级发展现代服务业。实施现代服务业全面提升行动，设立服务业发展专项资金，制订出台电子商务、商贸、交通物流、服务业总部经济等扶持政策。建设中马钦州产业园区北斗导航服务业基地、保税港区国际贸易产业集聚创新示范区等平台，争取华为云计算及大数据中心一期、信息港跨境数据中心等开工建设，加快建设中马大数据及智能基站天线研发产业基地、金达雷3D打印技术等项目。大力发展全域旅游，推进三娘湾创5A级景区、千年古陶城创4A级景区，建设茅尾海休闲度假区综合开发等重点旅游项目。大力发展休闲娱乐、家政服务等产业，组织开展城乡消费升级行动，培育新增“小上限”企业30家。

　　突出抓好产业链招商。继续实施招商引资三年行动计划。以产业链、供应链、创新链深度融合为方向，依托中石油、华谊等龙头企业重点突破树脂、合成橡胶、化纤等产业项目招商，延伸芳烃、烯烃及下游产业链，大力发展精细化工、化工新材料、特种化学品等产业。推进与中船、北港集团、华为、富士康等企业联合招商，重点引进装备制造、跨境电商、电子信息、物流配送等领域知名企业入驻。围绕打造海洋经济强市，积极引进现代海洋渔业、海产品精深加工、海洋生物制药等重点海洋产业项目，做大特色现代海洋产业。建立招商项目快速落地综合协调机制，主动靠前服务，梳理和解决项目落地障碍，促进更多签约项目实质性落户建设。

　　全力培植壮大财源。积极跟踪服务好石化、建筑、房地产、供电、烟草等重点税源企业，重点调整优化石化产品结构，增加应税产品量，同时推动炼油板块下游企业转型升级，提升石化产业税收效益。加快发展建筑业、房地产、交通运输业等对地方财力贡献较大的支柱产业，完善产业项目效益预评估机制，重点引进建筑、物流等总部经济，通过扩大产业经济规模产生更多经济效益和税收效益。强化税收征管，盘活财政存量资金，研究设立产业投资发展基金，发挥好财政资金对实体经济发展的引导作用和乘数效应。

　　强化项目建设要素保障。强化用地用海保障，积极争取新增建设用地用海指标，盘活用好存量建设用地用海。强化资金保障，完善政银企对接合作平台，培育发展各类新型金融市场主体，积极争取各类基金资金支持，推广应用应收账款融资服务平台，引导金融机构扩大对实体经济的信贷投放规模，增强金融服务实体经济能力。制订完善政府和社会资本合作配套政策，大力发展多层次资本市场，推进企业挂牌上市，多渠道拓宽项目融资渠道。统筹抓好煤、电、油、气、运等要素保障，确保420个总投资5000万元以上、年度计划投资1000万元以上重点项目顺利实施。

　　（二）抢抓南向通道建设重大机遇，推动构建“一带一路”有机衔接的国际陆海贸易新通道。充分发挥我市作为中新互联互通南向通道海铁联运重要节点的作用，主动融入南向通道建设，提升钦州的影响力、带动力，打造新的经济增长点。

　　实施港口集疏运建设攻坚。实施港口集疏运建设突破行动，启动建设钦州港东站铁路集装箱中心站、钦州港东站货场扩建二期等大型综合站场，加快推进南向通道物流基地、国际冷链中心等项目，集中破解港口配套设施瓶颈。加快实施钦州港火车站扩能改造、南钦铁路电气化改造等项目，建成钦港线与钦州港至大榄坪支线间联络线、钦州保税港区东卡口一期和铁路货场至码头“最后一公里”等海铁联运节点项目。加快金窝通用机场等项目前期工作，高标准开展疏港通道体系规划，加快建设大塘至浦北高速公路、兰海高速南宁经钦州至防城港段改扩建（钦州段）、南宁至钦州公路（南间至黎合江段）改扩建等工程，完成金鼓江大道扩建等工程，加快形成向北连通西南、中南等地区，南向连通新加坡等东盟国家、面向全球的陆海贸易大通道。

　　加快建设现代化集装箱干线港。围绕服务南向通道建设，及时调整港口、产业、城市等重点规划，尽快完成北部湾集装箱干线港规划。加大大榄坪、三墩等集装箱作业区开发力度，基本建成10万吨级集装箱双向航道一期同时加快二期建设，推进20万吨级集装箱航道及码头、大榄坪南9－11#泊位等大型航道、码头工程等前期工作，推动勒沟作业区15#泊位等一批码头验收开放。加密覆盖新加坡等国家或地区主要港口的集装箱直航航线，培育至欧美、非洲等远洋航线，推进与南向通道沿线节点城市合作建立“无水港”，积极发展国际中转集拼业务，尽快开通中欧集装箱班列。进一步清理和规范进出口环节收费，落实渝桂黔陇四省区市口岸通关一体化合作，推动建设北部湾中心海关，先行先试货物按状态分类监管等措施，扩大应用国际贸易“单一窗口”，推动实施“一票制、一口价”联运服务模式，争取设立进口毛燕、进境活牛等指定口岸，获批平行进口汽车试点，加快创建口岸优质服务示范区。

　　规划发展多式联运物流体系。围绕完善南向通道港航物流服务链，编制完善现代物流业发展规划，强化物流基础设施建设，优化物流节点空间布局，引进大型物流企业，培育多式联运龙头企业。推进中国－东盟中小企业贸易促进平台等建设，搭建线上线下物流信息平台，培育大宗商品交易市场。强化县域与钦州港的物流联系，建设陆屋临港产业园物流园、浦北农产品物流中心等县域重点物流节点。深化与国内外港航物流企业的合作，争取建设北部湾航运服务集聚区及钦州港、皇马综合物流园等现代物流集聚区，筹划设立北部湾航运交易所，培育发展航运服务、海事服务和中介服务，加快建设区域性物流企业总部基地。

　　（三）依托和发挥国家级平台优势，加快形成陆海内外联动发展新格局。主动融入“一带一路”建设，扎实推进市园一体化，充分发挥国家级平台聚集效应，增创开放合作竞争新优势。

　　推进“三区统筹”融合发展。树立“市园一体化”理念，建立健全国家级园区融合发展协调机制，整合优化国家级平台政策资源，启动建设自由贸易港的研究和规划，增强国家级平台对整体开放发展的带动作用。深入实施港产城联动发展战略，加快完善市区与中马钦州产业园区、钦州保税港区、钦州港经济技术开发区之间的快速公交网络，促进市区与港区、园区高效便捷互联互通，打造滨海新城、中马产业新城、钦州港新城三大港产城融合示范区。推进招商一体化，健全市园联合招商机制，增强招商整体竞争力。打破市园间的管理权限障碍，建立统一的政务服务和社会管理体系，实现市园间的要素资源共享。

　　发挥国家级平台集聚优势。支持中马钦州产业园区拓展“两国双园”合作领域，启动建设海峡两岸产业合作区钦州产业园、马来西亚创新城，加快建设鑫德利光电科技二期、凯利数码二期等项目，促进电子信息、生物医药、信息服务等优势产业集聚。提升钦州保税港区政策功能，建设广西国家进口贸易促进创新示范区核心区，完善标准厂房、冷链物流等配套设施，探索建立跨境电商业务监管新模式，规划建设中国－东盟（钦州）新能源汽车产业合作基地、跨境电商产业园，推进捷胜新能源汽车、金特安环保开式轮胎等项目加快建设，推动整车进口、酒类进口、加工贸易等核心产业和特色业务实现规模发展。

　　深化区域经济合作。拓展中国－东盟港口城市合作网络，完善中方秘书处运行机制，推进与泰国林查班港等缔结国际姐妹港，争取更多港口城市加入合作网络，全面建成海上搜救分中心等一期项目，推进中国－东盟港口物流信息中心数据对接、扩容建设，逐步实现港航信息互通共享。深化与南向通道沿线城市的合作，共建面向东盟的临海产业园。深入实施第二轮加工贸易倍增计划，建设钦州保税港区CEPA先行先试示范基地，重点承接港澳、东部发达地区电子信息、机电设备等高新技术产业，启动建设灵山、高新区等加工贸易产业园区，培育加工贸易龙头企业，扩大对外贸易和投资合作。

　　（四）全面深化改革创新，推动发展质量变革效率变革动力变革。以供给侧结构性改革为主线，创新有为，坚决破除一切不合时宜的旧观念和体制机制弊端，不断在改革创新中激发体制机制的强大活力。

　　深入推进供给侧结构性改革。全面落实“三去一降一补”，强化行业准入管理，保持重点去产能行业无违规新增产能，扶持企业革新技术装备，建立非国有“僵尸企业”处置机制，盘活制糖、铁合金等产业资源。坚持“房子是用来住的、不是用来炒的”，加快住房制度改革和长效机制建设，建立多主体供给、多渠道保障、租购并举的住房制度，积极推进房地产特别是非住宅商品房去库存，稳定房地产市场。完善金融支持实体经济发展的政策措施，营造良好金融生态，促进金融业健康发展，筑牢金融风险“防火墙”。开展“降成本政策落实年”活动，扩大直供电交易受益面，全面落实各项减税、免税和停免征部分政府性基金政策，加快推进政府性融资担保体系建设，切实减轻企业负担。继续实施园区基础设施三年大会战，建设完善标准厂房、路网、污水垃圾处理等产业配套设施和教育、医疗卫生、商业服务等生活配套设施，加快补齐园区基础设施短板。扎实开展城市管理、统计管理、交通运输等重点领域改革，稳妥推进事业单位改革。深化县域管理体制改革，推进扩权强县强镇，强化基层政府功能。推进社会办学、社会办医、社会养老等社会领域改革，降低社会资本进入公共服务和基础设施领域的门槛。

　　打造优质高效营商环境。不断深化“放管服”改革，完善政府部门权责清单，坚韧不拔地推进政府职能转变，强化绩效管理，重拳整治为官不为、懒政怠政。以市行政审批局高效运行为抓手，推进行政审批流程再造，进一步压缩审批时限，利用大数据技术推进“互联网+政务服务”，建立行政审批与监管协调联动工作机制，更好理顺钦州港行政审批局管理体制。继续完善“多证合一”登记制度改革，扩大全程电子化登记，开展“证照分离”试点，全面实施跨部门“双随机”联合检查，让企业和群众少跑腿，办事更加方便、更加快捷、更加舒心。开展“服务企业年”活动，大力构建“亲”“清”新型政商关系，实施投资环境满意度测评，健全项目建设“红黑榜”督查考评制度，设立投资环境投诉受理中心，聚焦企业发展的难点、痛点精准施策，及时解决项目推进、企业生产经营中遇到的困难和问题，破除制约企业发展的要素瓶颈，落实促进非公有制经济发展的各项政策，探索建立企业沟通机制，完善政府守信践诺机制，尊重企业和各类市场主体，激发和保护企业家精神，积极为企业发展创造更优环境、搭建更大舞台，以一流的营商环境构筑竞争新优势。

　　强化创新驱动发展。深入实施创新驱动发展战略、工业互联网创新发展战略，推进质量强市、标准强市、品牌强市，加快实体经济和数字经济融合发展。启动建设云计算产业园、新能源汽车产业园、国际医药创新园等新兴产业集聚平台，建成富士康医学影像云中心等大数据产业项目，大力发展“互联网+”经济，培育发展“制造业+”“旅游+”，促进新一代电子信息、新能源、生物医药等新技术、新业态、新模式与一二三产业融合发展。深入实施高新技术企业倍增计划，力争高新技术企业突破60家。实施发明专利双倍增计划，鼓励社会主体投身创新创业，支持企业、高校、科研院所、创客合作搭建产学研合作平台，激发和释放全社会创造潜力。新发展一批工程技术研究中心、重点实验室、企业研究院，争取建成市高新技术产业服务中心，筹备申报国家级科技企业孵化器。继续实施引进领军型创业人才、非公有制企业高层次人才等引才项目，培养造就高水平科技领军人才和创新团队，带动形成“大众创业、万众创新”的生动局面。

　　（五）统筹推进城乡一体化发展，建立更加有效的区域协调发展新机制。实施区域协调发展战略，做强县域经济，加快推进新型工业化城镇化，培育县域发展新优势。

　　建管并重提升城市品位。积极融入北部湾城市群建设，加强城市风貌建设，编制城市总体设计、钦州港区总体规划。加快推进滨海新城开发，开工建设嘉兴街地下综合管廊、茶山江大街等项目，加快安州大道建设，打造提升白石湖兰亭街商业圈、沙井岛旅游集散基地、滨海岸线景观带等城市功能区，打通白石湖片区、沙井岛片区、辣椒槌片区路网。推进“一环六横六纵七通道”主干路网建设，贯通金海湾大街以北的环路、通富路等道路，进一步完善城市路网，有效解决市区停车难、交通堵点等问题。加大对城中村及老城区的改造建设力度。抓好排水系统改造工程，建成河东污水处理厂提标改造等工程。加快实施郁江调水工程、大马鞍水库扩容工程，保障城区用水安全。实施创建国家生态园林城市三年攻坚行动，加快江滨公园钦江西岸、子材公园等公园景观以及大榄江湿地公园建设，加大河西片区绿化改造力度，增加城市绿量。探索引入共享单车、共享汽车，完善城市慢行系统，解决群众出行“最后一公里”难题。加快推进国家智慧城市试点，实施智慧社区、智慧健康等项目，尽快建成“数字城管”。开展宜居城市创建活动，进一步提升市民获得感、幸福感。

　　加快推进城镇化建设。实施大县城战略，加快灵山、浦北新型城镇化示范县建设，推进钦北新城建设，全面建成县县通天然气工程。加大陆屋全国特色小镇建设力度，力促龙门红椎小镇、坭兴陶小镇、大寺飞翔小镇、钦州港海上牧场浪漫小镇等创建成为国家或自治区级特色小镇。开工建设浦北至北流高速公路，推进灵山县绕城公路、滨海公路环茅尾海支线九鸦至茅岭段前期工作，加快石埇至钦州二级公路、新城至小董二级公路等建设，按照“四好农村路”要求加快完善村屯公路网络，推进城乡公交一体化，规划建设中心城区至灵山、浦北县城的快速通道，构建一小时城市生活圈。推进县域基本公共服务均等化，深化户籍制度改革，引导农业转移人口有序市民化，力争常住人口城镇化率达到40%。

　　打造县域经济升级版。树立“全市一盘棋”思想，建立利益分享机制，鼓励县区打破行政地域界限开展联合招商，提升县域整体竞争力。实施县域工业提升工程，完善提升灵山工业区、浦北工业集中区、钦北皇马工业园、钦南金窝工业园，申报创建自治区级经济技术开发区，支持建设灵山陆屋机电产业园、浦北林木循环经济产业园、钦南食品科技园、钦北中医药健康产业园等县域特色产业园，大力发展电子信息、机电、林木深加工、生物医药等县域特色产业集群。推进全国电子商务进农村示范县项目建设，支持实体商业创新发展，撬动县域消费升级。推进八寨沟、五皇山建设成为国家生态旅游示范区，龙武庄园、石祖生态园、白石湖公园、九佰垌等创建4A级景区，大力发展健康养老、生态旅游、休闲农业，擦亮“世界长寿之乡”等金字招牌。

　　（六）大力实施乡村振兴战略，加快推进农业农村现代化。始终用乡村振兴来统领“三农”工作，坚持农业农村优先发展，遵循乡村发展规律，加快补齐农业农村现代化这条短腿，着力推进农业强、农村美、农民富。

　　深化农业农村改革。深入推进农业供给侧结构性改革，推进农业农村“放管服”改革，破除一切束缚农民手脚的不合理限制和歧视。深化粮食收储制度改革，稳定粮食产能。落实“三权分置”，全面推进农村土地承包经营权确权登记颁证工作，开展宅基地和集体建设用地确权登记改革。健全县（区）、镇、村三级农村土地流转服务和管理机制，加快发展土地流转型、服务带动型等多种形式适度规模经营。构建现代农业产业体系、生产体系、经营体系，培育一批新型农业经营主体和新型职业农民，发展一批农民专业合作社和家庭农场。继续推进集体林权制度、小型水利工程管理体制等改革工作。

　　大力发展农业产业。把大力发展农村生产力放在首位，支持和鼓励农民就业创业，用有奔头的产业、有吸引力的职业留住农村年轻人口，拓宽增收渠道，为乡村聚人气、添活力。实施现代特色农业示范区创建增点扩面提质升级三年行动，提升发展灵山百年荔枝、浦北佳荔水果产业、钦南虾虾乐、钦北那蒙特色苗木、钦州港大蚝养殖等一批现代特色农业示范区，争取创建更多自治区级核心示范区。推进“双高”糖料蔗基地建设。继续创建全国、全区休闲渔业品牌，建设国家级海洋牧场示范区，大力发展大蚝、荔枝、火龙果、百香果等特色农产品加工业。实施质量兴农战略，加强特色农产品品牌建设，提升钦州大蚝、钦州富硒米、灵山荔枝、浦北芋头等特色农产品品牌价值。大力发展智慧农业、观光农业、体验农业、创意农业等新产业、新业态，加快农村一二三产业融合发展。

　　共建共享美丽乡村。深入开展“美丽钦州·宜居乡村”活动，推进产业富民、服务惠民、基础便民、文化乐民等专项活动。把公共基础设施建设的重点放在农村，加快灌区节水改造、便民候车亭、中心村电网改造等农业农村基础设施项目建设，实现全市村级综合服务中心建设全覆盖、宽带信息网络全覆盖，切实改善农村群众生产生活条件。着力推进“厕所革命”，开展新一轮农村环境整治，全面完成农村垃圾专项治理两年攻坚行动，努力提升农村环境卫生水平。重视乡村规划，强化古村落保护，大力加强农村精神文明建设，建设美丽宜居乡村。

　　（七）贯彻绿色发展理念，打造山清水秀生态美靓丽名片。坚持“绿水青山就是金山银山”，坚持节约资源和保护环境的基本国策，把良好生态环境作为最普惠的民生，打好生态环保攻坚战，坚定走生产发展、生活富裕、生态良好的文明发展道路。

　　强化生态系统保护。加大耕地保护力度，开展全国第三次土地调查，做好永久基本农田划定工作，重视耕地丢荒问题。启动建设生态林业科技创新示范区，抓好公益林管护和红树林保护，大力开展植树造林，加快建设国家森林城市，力争森林覆盖率保持在54.2%左右。全面推行“河长制”，着力构建江河湖库管理保护新格局。推进饮用水水源保护地方立法，实施农村饮水安全巩固提升工程，确保群众喝上“安全水”“放心水”。加快生态文明示范区建设，开展生态保护红线划定，推进生态补偿试点工作，创建一批自治区级生态村，优化生态安全屏障体系。推动设立钦州中华白海豚自然保护区，增强海洋资源保护意识，严厉打击非法捕捞等破坏海洋生态环境的行为，让钦州成为中华白海豚繁衍栖息的最美家园。

　　加强环境问题整治。统筹山水林田湖草系统治理，健全联防联控、督查考核等机制，加大生态环境风险隐患排查整治，严格落实环境保护和节能减排“一岗双责”，深入抓好中央环保督察、国家海洋督察等反馈意见整改落实。继续开展第二次全国污染源普查，深入实施大气、水、土壤污染防治计划，组织开展蓝色海湾整治行动，推进钦江、大榄江、南流江等流域环境综合整治和东西干渠等城市黑臭水体专项整治。加强固体废弃物和垃圾处置，构建政府为主导、企业为主体、社会组织和公众共同参与的环境治理体系，着力提升生态环境质量。

　　推进绿色低碳发展。加大节能减排降碳宣传，继续开展绿色低碳创建活动，推进资源全面节约和循环利用。改革环境保护管理体制，抓好排污费改税工作，建设市级层面碳排放权交易市场。推动低碳循环生产，培育一批循环经济示范企业、绿色工厂，加快实施茅坡社区省级低碳社区试点，开展企业碳排放核查。大力发展风电、太阳能发电等新能源产业，以及生态种养、生态旅游等绿色产业，提高清洁能源比重，促进生态优势向经济优势转化。

　　（八）坚持以人民为中心的发展思想，不断满足人民对美好生活的向往。抓住人民最关心最直接最现实的利益问题，尽力而为，量力而行，扎实做好保障和改善民生工作，不断增强人民群众的获得感、幸福感和安全感。

　　致力攻坚抓脱贫。坚持扶真贫、真扶贫，强化党政一把手负总责，进一步压实扶贫责任，健全扶贫动态调整帮扶机制，鼓励引导社会力量参与扶贫，激发贫困人口内生脱贫动力，确保全面小康路上一个都不少。加快推进贫困村基础设施建设，进一步改善贫困村基本生产生活条件。加快建设中马特色扶贫小镇项目。突出抓好产业扶贫开发，实施产业扶贫示范小区带动扶贫脱贫试点，发展壮大薄弱村空壳村集体经济，推进就业扶贫车间建设，创新建立扶贫户参与机制和受益机制，探索贫困户资产性收益增收路子，力争贫困户人均资产性收入年均增幅达到20%以上。扎实推进金融扶贫、旅游扶贫、低保助力、健康扶贫、电商扶贫，推行建档立卡贫困家庭学生15年免费教育，统筹做好易地扶贫搬迁后续产业、就业扶持，实现年度脱贫减贫目标。

　　持之以恒保民生。继续实施为民办十件实事工程。实施就业优先战略和更加积极的就业政策，开展全方位公共就业服务，实现更高质量和更充分就业。实施劳动合同制度，推进企业建立集体协商制度，积极构建和谐劳动关系。全面实施全民参保，完善养老、医疗保险制度，强化社会救助，织密民生保障网。积极发展养老服务业和老龄产业，健全农村留守儿童和妇女、残疾人关爱服务体系。全面建成覆盖全民、城乡统筹、权责清晰、保障适度、可持续的多层次社会保障体系，有效保障困难群众基本生活。

　　协调发展社会事业。实施广西教育提升新三年行动计划，优化高中教育资源布局配置，完善现代职业教育体系，办好学前教育、特殊教育和继续教育，支持和规范社会力量兴办教育，引进中央民族大学附中创办分校，全面推进义务教育学区制改革和集团化管理，加快完成“全面改薄”任务，培植优质教育资源，确保各县区通过义务教育均衡发展“国检”。着力推动钦州学院升格为北部湾大学。全面深化公立医院综合改革，稳步推进医疗联合体和家庭医生制度建设，不断完善分级诊疗制度，探索建立现代医院管理制度。做好人口和计划生育工作，创造良好人口环境。大力实施文化惠民工程、文化精品工程，加强非物质文化遗产传承保护工作。组织开展纪念冯子材诞辰200周年系列活动。加快发展特色文化产业，重点建设坭兴陶文化创意产业园、小江瓷文化产业园等特色文化产业基地，继续推进创建中国历史文化名城和坭兴陶烧制技艺申报世界非物质文化遗产工作。深入推进健康钦州建设，实施全民健康素养促进行动计划，加快发展体育事业和体育产业，办好世界沙滩排球巡回赛钦州公开赛等国际赛事。实施永福大街、蓬莱大道白改黑工程和小街小巷提升工程，持续推进创建全国文明城市工作，培育和践行社会主义核心价值观，加快诚信体系建设和志愿服务制度化。充分发挥工会、共青团、妇联等群团组织的作用，继续做好民族、宗教、外事、侨务、对台、人防、打私、双拥共建、优抚安置、民兵预备役和边海防等工作，促进统计、档案、哲学社会科学、方志、文史事业全面发展。

　　筑牢社会治理屏障。实施食品安全战略，做好重大疾病防控，大力创建国家卫生城市、国家食品安全示范城市，落实食品药品安全监管“四个最严”，让人民吃得放心。强化公共安全意识，健全安全生产责任体系、安全风险分级管控和隐患排查治理体系，抓好森林防火和消防安全工作。切实强化信访调处工作，完善社会矛盾纠纷多元化解机制，加快立体化信息化社会治安防控体系建设，依法打击和惩治黄赌毒黑拐骗等违法犯罪活动，构筑全民共建共治共享的社会治理格局。加强基层治理体系建设，发挥社会组织作用，实现政府治理和社会调节、居民自治良性互动。做好气象灾害预报预警、防汛防台抗旱各项工作，完善应急和防灾救灾机制，加快创建国家防震减灾安全示范城市，保障群众安居乐业、社会安定有序。深入开展“七五”普法工作，在全社会形成浓厚法治氛围。

　　三、建设人民满意的服务型政府

　　新时代昭示新使命，新使命需要新担当。我们要坚持把讲政治放在首位、作为根本，牢固树立“四个意识”，坚决维护以习近平同志为核心的党中央权威，自觉在思想上政治上行动上同党中央保持高度一致，努力建设人民满意的服务型政府。

　　（一）坚持依法行政。带头尊崇法治、敬畏法律、依法办事，牢固树立职权法定观念，坚持法无授权不可为、法定职责必须为。坚持科学决策、民主决策、依法决策，广泛听取各方面意见包括批评意见。深化行政执法体制改革，加强行政复议规范化建设，完善政府法律顾问制度。依法接受市人大及其常委会监督，自觉接受人民政协的民主监督，主动接受社会和舆论监督，认真听取人大代表、政协委员、各民主党派、工商联、无党派人士和各人民团体的意见，办理好人大代表建议和政协提案。

　　（二）坚持为民施政。坚持人民主体地位，把执政为民理念融入到感情里、体现到政策中、落实到行动上，做到为民做事不嫌小、解民之忧不嫌多、问民疾苦不嫌杂。为官避事平生耻，为民服务敢担当，要深入实际调查研究，善于发现问题、敢于提出问题、勇于解决问题，以实际行动积极回应群众期待，满足群众需求，维护群众利益，让全市人民得到更多实惠。

　　（三）坚持廉洁从政。认真履行全面从严治党政治责任，开展“不忘初心、牢记使命”主题教育活动，加强和规范党内政治生活，严格遵守政治纪律和政治规矩。全面落实党风廉政建设主体责任和“一岗双责”，进一步贯彻落实中央八项规定精神，驰而不息纠“四风”转作风。推进国家监察体制改革，健全廉洁风险防控体系，加强行政监察、审计监督和财政监管，筑牢制度“篱笆”。集中财力办大事，确保“三公经费”只减不增。始终保持反腐高压态势，开展扶贫领域腐败和作风问题专项治理，坚决从严查处群众身边的腐败问题和不正之风，进一步树立干净干事、清正廉洁的政府形象。

　　各位代表！中国特色社会主义进入了新时代，钦州发展也开启了新征程。回首来路，我们从全市人民自筹资金起步，建大港、兴产业、筑平台、造新城，以“一天填海造地40亩、七天建成一层楼”的“钦州速度”，创造了一个又一个令人惊叹的“北部湾奇迹”。钦州有今天令人自豪的发展成就，全都是广大干部群众团结一致、实干苦干创造出来的。面向未来，钦州的发展任务更加繁重，挑战更加巨大，南向通道建设的历史机遇千载难逢，更需要全市各级领导勤政为民、以上率下，一棒接着一棒干；更需要全市广大干部勤勉务实、勇于担当，撸起袖子加油干；更需要全市各族人民勤苦劳作、共同奋斗，甩开膀子大胆干！天道酬勤，只要我们努力了、付出了，就一定能实现“最初的梦想”。

　　让我们更加紧密地团结在以习近平同志为核心的党中央周围，以习近平新时代中国特色社会主义思想为指引，在市委的坚强领导下，不忘初心，牢记使命，同心同德，开拓进取，努力完成今年经济社会发展各项目标任务，为把钦州建设成为“一带一路”南向通道陆海枢纽城市，奋力谱写新时代钦州发展新篇章而不懈奋斗！
